# Exploring Behavioral Creativity of a Proactive Robot

**DOI:** 10.3389/frobt.2021.694177

**Published:** 2021-11-26

**Authors:** Sera Buyukgoz, Amit Kumar Pandey, Marine Chamoux, Mohamed Chetouani

**Affiliations:** ^1^ SoftBank Robotics Europe, Paris, France; ^2^ Institute for Intelligent Systems and Robotics, CNRS UMR 7222, Sorbonne University, Paris, France; ^3^ Socients AI and Robotics, Paris, France; ^4^ BeingAI Limited, Hong Kong, Hong Kong SAR, China

**Keywords:** proactive robot, creative behavior, self-initiated behavior, human–robot interaction, social robot

## Abstract

Creativity, in one sense, can be seen as an effort or action to bring novelty. Following this, we explore how a robot can be creative by bringing novelty in a human–robot interaction (HRI) scenario. Studies suggest that proactivity is closely linked with creativity. Proactivity can be defined as acting or interacting by anticipating future needs or actions. This study aims to explore the effect of proactive behavior and the relation of such behaviors to the two aspects of creativity: 1) the perceived creativity observed by the user in the robot’s proactive behavior and 2) creativity of the user by assessing how creativity in HRI can be shaped or influenced by proactivity. We do so by conducting an experimental study, where the robot tries to support the user on the completion of the task regardless of the end result being novel or not and does so by exhibiting anticipatory proactive behaviors. In our study, the robot instantiates a set of verbal communications as proactive robot behavior. To our knowledge, the study is among the first to establish and investigate the relationship between creativity and proactivity in the HRI context, based on user studies. The initial results have indicated a relationship between observed proactivity, creativity, and task achievement. It also provides valuable pointers for further investigation in this domain.

## 1 Introduction

Robots are becoming more and more a part of our lives. We encounter robots in our houses as assistants, at schools as tutors or peers, and at marketplaces as guides or shopping assistants. Robots appear not as tools but as social agents with a voice and a mind in our daily lives. Robots are producing behaviors that are intended to be supportive or helpful to the user. However, there is a need to investigate how such behaviors might be related to the users’ expectations, causing some kind of confusion, or even related to the user’s creativity. This will help in crafting the right level of behavior and suggestions that the robot should be providing. Robots can have different strategies to interact, such as *reactive*, when it acts only if there is a demand from the user, or *proactive*, where it acts even if there is no explicit request from the user. This study focuses on the proactive behavior of a robot with the intention to support the user in performing a task.

In organizational psychology, proactive behavior is defined as anticipatory, self-initiated, change-oriented, and future-focused behaviors (Grant and Ashford, 2008). Proactivity is described as a process of individuals influencing their environments (social, non-social, and physical) (Bateman and Crant, 1993) by intentionally taking initiatives (Bateman and Crant, 1999), by utilizing the combination of knowledge, perception, and ability to predict others’ actions and consequences (Tomasello et al., 2005). Most human–robot interaction (HRI) studies are based on this definition from organizational psychology to define proactive robot behavior where robots must be anticipatory, self-initiated, and change-oriented toward future changes (Peng et al., 2019). In this study, we analyze the notion of proactive action as perceived from the user’s perspective. Therefore, any act by the robot needs to be fulfilling the following two conditions for it to be perceived as proactive action (as opposed to reactive action) by the user: 1) there is an anticipation of the future situation. This can be either by a human controller or autonomously by the reasoning mechanism. 2) Based on the anticipation, if the robot is behaving without any explicit request from the user, it is self-initiated behavior of the robot from the user perspective. Again, such acts by the robot are instantiated either by a remote operator or autonomously. This situation is enough for us to perform our studies of proactive behavior from the user perspective. We are interested in understating the effect of such behaviors on the user, not how such behaviors should be created.

On the other hand, there is a notion of creativity, which refers to the novel product of value (Weisberg, 1993) or a person who expresses novel thoughts (Csikszentmihalyi, 2009). Being creative is the ability to change existing perspectives (Goncalo, 2019). In that sense, to be creative and proactive, both carry the similar notions of anticipatory, self-initiated, and future-driven behaviors. Therefore, in one sense, proactivity and creativity are highly coupled. Creativity, as the ability to produce novel ideas, is argued to be a necessity for proactive behaviors, and proactive personality is positively associated with creative behaviors (Joo and Bennett, 2018). In order to be creative, it is essential to have the ability to view things from different perspectives and generate new possibilities or alternatives in a unique way (Franken, 1994).

The majority of the examples we saw in robotics use interaction patterns to support users’ creativity. Robots adopt the role of either a supportive agent that facilitates the user’s creativity (Elgarf et al., 2021; Alves-Oliveira et al., 2019) or a creative peer that is collaborating with the user on a creative task (Law et al., 2019; Lin et al., 2020; Hu et al., 2021). In that sense, all of the examples put creative thinking of the user as an aim of the robot. Even some researchers claim that there is a positive effect of robot usage in education on a child’s creative thinking (Ali et al., 2019).

In reality, life is not focused on creative thinking, even to the extent that educationalists complain that the current education system is blocking creative thinking. Ken Robinson stated in his TED talk that school kills creativity. The current education system depends on convergent thinking, asking for the answer to a question, rather than divergent thinking, asking how to reach that answer (Ritter et al., 2020). If we think about the task-based robotics system, which is popular in robotics systems, how could they cope with supporting the user’s creativity?

In our study, on the proactive side, we focus on the robot being proactive toward the user who is completing the task of cooking recipes. The robot exhibits proactive actions by predicting what users try to achieve without the user asking for information or support and instantiates a set of verbal communications with the user. On the creativity side, we focus on the two aspects of creativity: creativeness observed by the user in the robot proactive behavior and the creativity of the user while leading toward a task of reaching a cooking recipe.

Our motivation is to understand the effect of proactive behavior and the relation of such behaviors to the two aspects of creativity discussed above. This study aims to present the results of a set of pilot studies of different behaviors of the robot. The idea is to understand and explore the limitations and pointers for conducting a full-scale research project. To our knowledge, it is the first study of its kind, which is trying to explore the connections between observed proactivity, creativity, and task achievement, in a setup of users with mixed backgrounds. The initial results have indicated some interesting relations among various attributes. At the same time, it is hinted that it would be too early to draw a definite guideline and conclusive relations about the optimum behaviors of the robot. Nevertheless, our findings suggest various parameters, which need further investigation when such social robots will serve people in day-to-day activities, where a series of actions are needed.

## 2 Background

Creative thinking is defined as a skill that produces novel and valuable ideas (Sternberg, 2010). It is a way to consider things from a different perspective, be creative, and have a different look at daily life problems. It depends on the knowledge of the individuals. There is no prior way to define creativity. Although the creative contributions are classified under eight headings (Sternberg, 2010), evaluating the amount of creativity is not clearly defined. Some types could have greater amounts of novelty than others. Creative thinking is not the equivalent of divergent thinking. However, divergent thinking tests can be used for estimating creative thinking (Runco, 1993). Divergent thinking is defined as the ability to produce diverse ideas (Runco, 1993).

Producing creative ideas is affected by the environment. However, creativity requires a moderate level of focus. Therefore, when a person is interrupted or the attention process gets disturbed, it might affect the creativity performance (Woodman et al., 1993; Wang et al., 2014). In that sense, potential interruptions by the robot to the human performing a task, which might occur because of the proactive interactions, might have effects on the creativity of the person. Furthermore, interruptions might also come from the environment and/or other agents during the interaction.

Several human–human interaction studies are providing an interesting understanding of the relationship between receiving interruptions and being creative. Different types of moods on the interruptions are highly studied in the cognitive science domain—most of them are based on variations of the tone of the interruptions. Studies have shown the positive effect of a positive mood of the interruptions on creativity (Baas et al., 2008; De Dreu et al., 2008) to generate new ideas. Another study shows the strong relationship of receiving different types of interactions and being creative. Studies have shown the positive effect of positive feedback on creativity to generate new ideas (George and Zhou, 2007; Gong and Zhang, 2017). The positive interruptions carry combinations of positive feedback—validating or praising the user—and constructive feedback—question users’ actions and lead them to think about the solution they find (Gong and Zhang, 2017). The effect of negative feedback in connection with the frequency of positive feedback is also studied. For example, if the level of positive feedback is high, then negative feedback positively affects the creativity performance (George and Zhou, 2007; Gong and Zhang, 2017). Different studies are highlighting the effects of interruptions’ tone, type, and frequency to be creative.

### 2.1 Proactivity in Robotics

The previous aggregated definitions of proactivity align with many of the recent studies in robotics for defining a robot’s proactivity as the anticipatory action initiated by the robot to impact itself or others (Peng et al., 2019). It is defined as acting before it is requested (Ujjwal and Chodorowski, 2019). The proactivity of robots is studied in different implementation methods by using different initiations. The most related ones are as follows: 1) *anticipating user needs*, which is when the robot understands the user’s needs and offers its support (by acting or interacting) for clarifying confusion (Pandey et al., 2013) or for providing suggestions (Peng et al., 2019; Baraglia et al., 2016; Grosinger et al., 2016; Myers and Yorke-Smith, 2007; Bader et al., 2013; Zhang et al., 2015; Ujjwal and Chodorowski, 2019), 2) *for anticipating* possible plan failure and plan repair (White et al., 2017), 3) *for preventing* future hazards (Bremner et al., 2019), 4) *improving the robot’s knowledge & seeking information*, such as the robot asking the user for validation or the robot asking the user to identify gaps in the robot’s knowledge (Lemaignan et al., 2010) (Moulin-Frier et al., 2017), 5) *seeking engagement and interaction,* such as proactively seeking the user for interaction (Garrell et al., 2013) or continuing interaction (Liu et al., 2018), 6) *adapting to the user*, such as the user’s action while working together (Awais and Henrich, 2012), following the speed of the robot while considering constraints that the user needs to meet (Fiore et al., 2015), or considering the user’s habits and arranging the robot’s action not to become annoying (Rivoire and Lim, 2016) or enacting humanlike behaviors while reaching the object (Cramer et al., 2009; Han and Yanco, 2019), and 7) *adapting robot roles*, such as changing the robot from the leader to the follower during cooperative manipulation tasks (Thobbi et al., 2011; Bussy et al., 2012).

There are various modalities to exhibit proactive behaviors, from manipulation to verbal suggestions. However, while considering the limitations of social robots, it is already challenging to manipulate the shared environment physically since social robots’ manipulation capabilities are not as precise as those of collaborative robots or industrial lightweight robots. On top of that, some HRI studies show that humans are more accepting of the robot’s proactivity when the robot includes the human in the decision instead of the robot applying the decision itself (Kraus et al., 2020). Therefore, to simplify the complexity of the experiment and to avoid imposing a decision, we chose to instantiate communicative actions, thus limiting other modalities to make the robot proactive.

### 2.2 Creativity in Robotics

Creativity is studied in different areas of robotics. The majority of the task definitions are inspired by figural and verbal creativity. For instance, users are assigned to tell a story (Elgarf et al., 2021) or draw a figure (Alves-Oliveira et al., 2019). Robots produce behaviors for supporting the user’s creativity. Robots present a collaborative behavior where they study social aspects and engagements of robots with turn-taking principles. The robot’s role depends on supporting the user’s creative behavior, where the robot is most likely asking the user to think about their decision and lead them to be creative. As in the example of Kahn’s Zen Garden (Kahn et al., 2016), the robot follows a pattern of interaction to foster the user’s creativity.

The robot’s creativity is also explored in various studies. For example, the study of human–robot collaborative design (Law et al., 2019) aims to facilitate creativity of the user and be creative as a robot. Both the robot and the human are playing creative roles in the task. So, the robot supports the user and tries to be creative in the decision process to design a pattern. In their study, the robot’s and the human’s creativity share the same definition of being unexpected, novel product creation. Meanwhile, in the study of co-creativity (Lin et al., 2020), the focus is on facilitating the robot’s creativity by getting feedback from the user while collaborating to draw a figure. The main point is to lead the robot to more creative outcomes (Hu et al., 2021), since the robot mostly creates ideas during collaboration.

The creativity process requires an environment of help or individual effort to develop ideas for self-initiated projects (Apiola et al., 2010). In this sense, a robot’s creativity depends upon the robot’s ability to produce helpful information. In this study, the creativity of the robot focuses on the definition of creating useful help. This created help is communicated to the user without being asked for it and is hence used as a proactive robot behavior. As such proactive behaviors are interrupting the users, they have a potential effect on the creativity of the user as well.

Thus, this study explores the behavioral aspects of creativity, inspired by our previous study of creativity and proactivity (Buyukgoz et al., 2020), which hinted about the possible relationship between these two aspects, and also suggested the need to investigate the duality of creativity: *creativity of the robot* and *creativity through the robot*. More specifically, in that work, we developed a study to experiment with the robot’s proactive behavior when there is no task explicitly assigned. The behavior occurs as a set of verbal interruptions toward the users as a result of anticipation of the situation. The study was for understanding how the robot’s proactive behavior is perceived as a creativity of the robot and its effects on the user’s creativity in the HRI scenarios. Although, as discussed earlier, the frequency of interruptions also has a role to play in creativity, we also instantiated different levels of proactive interactions to explore the effects.

## 3 Design Overview

This study explores the behavioral aspect of creativity during HRI using a robot as a proactive agent, creatively engaging in a task performed by the user and generating suggestions as communicative behavior to guide the user while achieving their intended goal. In this regard, *proactive behavior* of the robot is defined as instantiating behavior for suggesting the users, where the robot’s intervention is not necessary or not requested by the user. We have considered a common home scenario task—cooking a recipe—that is explained below.

### 3.1 Task Definition

We have chosen a task in which the user could be creative and would not necessarily need the robot’s help to complete the given assignment. Generating cooking recipes can be seen as a creative task, in which the users could converge toward different recipes by using a similar set of ingredients.

Most likely, users have differing knowledge about the typical recipe. That is why users were provided with seven recipes with the dish’s name and ingredients to set the expected ground for the cooking recipe scenario. Each dish has six different ingredients. The recipes are different. However, each dish shares one or more ingredients with other dishes. In total, twenty-two ingredients were given in alphabetical order from different categories: proteins; chicken, foie-gras, ham, lardon. Vegetables; garlic, pumpkin, spinach, truffle, onion, pepper, nutmeg. Dairy products; milk, cheese, butter, eggs. Processed stuff; bread, barbecue sauce, stock, wine. Sugary stuff; sugar, honey. Basics; flour. Typical French recipes such as quiches, soups, and toasts were chosen to eliminate the hassle of learning new recipes. The whole task is divided into two phases to first study the effect of proactive behavior for a predefined recipe and then give the user a chance to be creative by eliminating the predefined recipe. With each user, the experiment starts with Phase 1 and continues with Phase 2.

Phase 1: a dish is assigned to the user. The participant should select the exact ingredients for the given recipe. Thus, the participant and the robot both know which recipe is targeted.
Phase 2: the user is asked to create a dish by using the given ingredients. The robot does not know about the target dish.

### 3.2 Design of the Proactive Behavior

**FIGURE 1:**
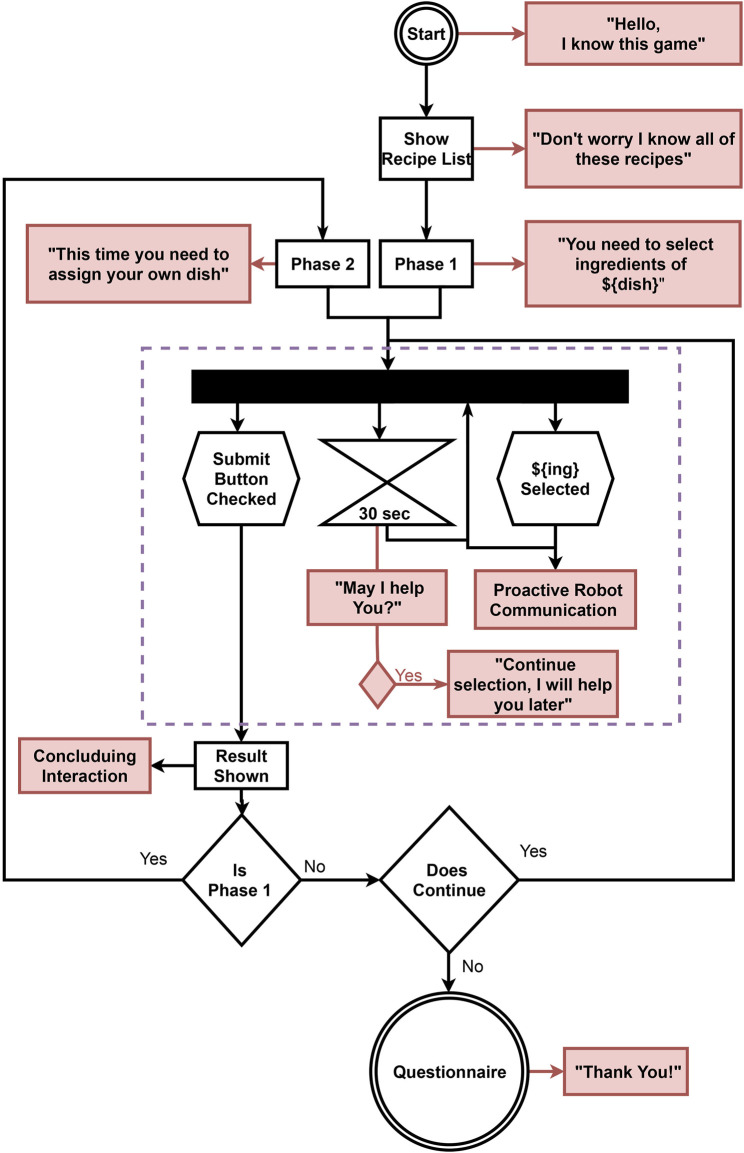
Activity diagram of interaction flow indicates the actions of the task, the robot, and the user. Black rectangle boxes represent the pages of the task and diamond boxes show the decisions of the task for general flow of phases. Robot responses are indicated with red boxes. Red boxes with black arrows represent the sentence that will be created. The user’s actions are represented with hexagon boxes.

**FIGURE 2:**
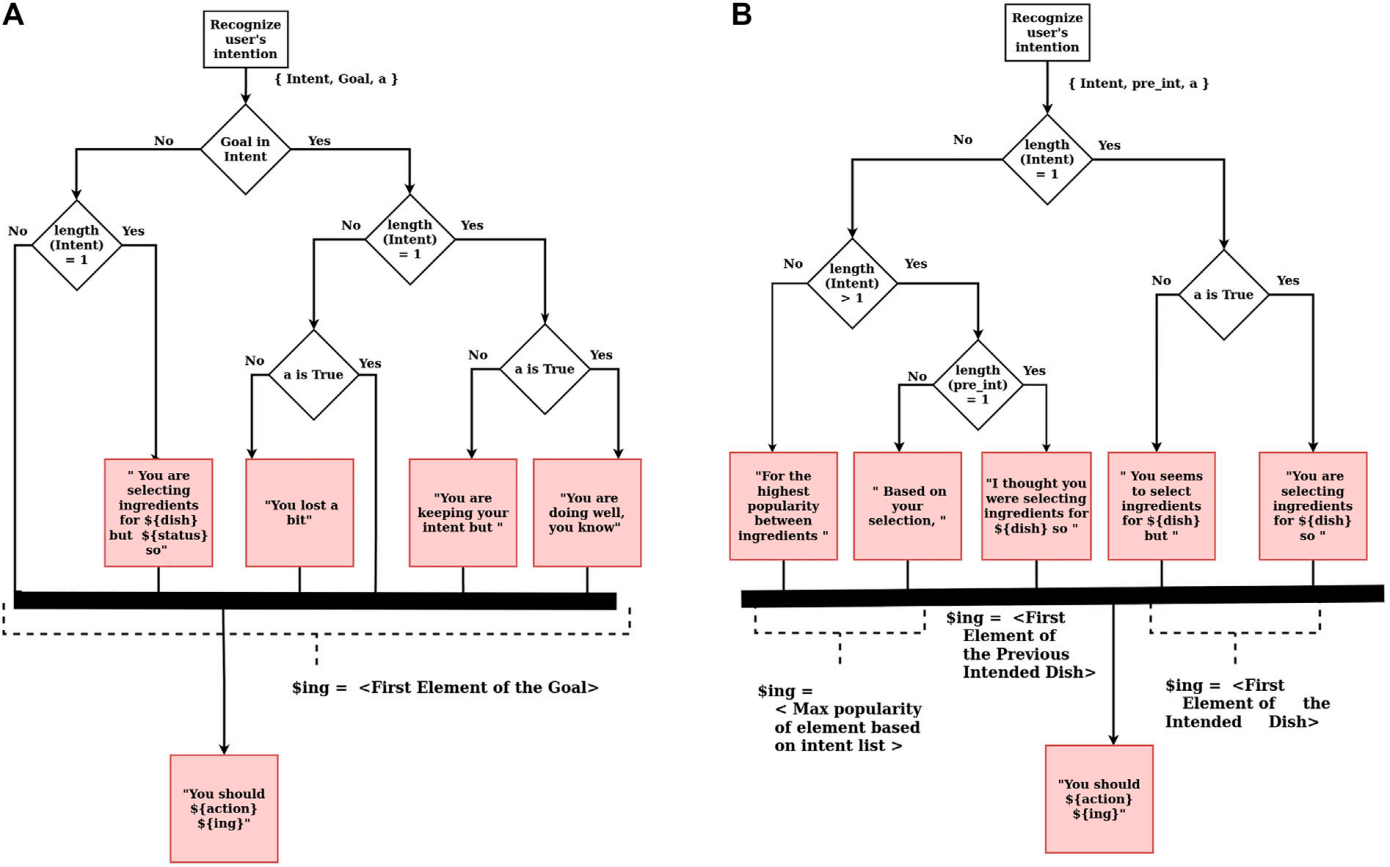
Diagram of proactive robot communicative is showing how proactive suggestions are initiated. It is starting with recognizing the user’s intention. The list of the user’s intent passes to the reasoning mechanism which is based on layers of rules. Red boxes show the robot response. In boxes, the template is given to create a sentence. Between curly brackets of *$*{…} filled with symbolic representation of action (${action}), object–ingredient (${ing}) or dish (${dish})–or the result of system (${status}). Each box concatenates to each other on the road to create proactive behavior of the robot.

In this study, the proactive behavior of the robot uses shared principles of creativity and proactivity. Those principles consist of 1) being anticipatory, based on a particular state, 2) self-initiated, producing proactive suggestions without them being explicitly demanded by the user, and 3) future-driven, trying to converge toward the needs of the goal. A rule-based system is developed for the robot to instantiate verbal suggestions depending on the user’s task. Rules are selected based on the task’s needs and the understanding of intention recognition. With the help of the rules, reasoning occurs to instantiate the parameters of the proactive suggestions. The robot’s knowledge and this reasoning result are proactive robot communication (see Figure 1). The decision flow of instantiating the proactive robot communication (aka proactive suggestion) is shown in Figure 2.

The set of rules varies according to the need of the task. The tone of the proactive behavior slightly differs depending upon whether there is a target dish assigned by the system (Phase 1) or not (Phase 2). The decision-making processes to instantiate the proactive interaction, for Phase 1 and Phase 2, are shown in Figures 2A,B, respectively. In both cases, instantiating the proactive robot communication starts with intention recognition of the user actions. Intention recognition is the recognition of the user’s target dish by interpreting the robot’s knowledge of the dishes and the ingredients that the user has selected so far. The recognition process is a simple rule-based mechanism that checks how close the user is to achieving one goal. The user’s intention is based on the least number of ingredients left from the set of known dishes. The intention is either the list of dishes or a single dish, depending on the situation of the selected ingredients so far. The user is also free to move away from the set of dishes that the robot knows and create their own dish by selecting a new list of ingredients. The user is assumed to be reliable and collecting ingredients to complete a dish. The user willingly performing a faulty behavior to deviate the intention recognition is not handled in this recognition mechanism. In the beginning, the intention of the user is all the dishes that the robot knows. Then, the recognition mechanism updates the user’s intention for every change in the state (adding or removing an ingredient). Respectively, the system initiates the new proactive suggestion.

Different sets of rules are used to instantiate the sentences’ templates, depending on whether the goal is assigned (phase 1) or not (phase 2). In phase 1 (see Figure 2A), it is crucial to accomplish the assigned dish by selecting the exact ingredients of the target dish. That is why intention recognition responds to each change in the state by updating the list of intentions. Updating the intentions triggers the process of instantiating the sentences’ templates. The next step of “*Goal in Intent*” (as shown in Figure 2A) is to check if the targeted dish (which is the goal as shown in Figure 2A) is part of the intention list or not. This reasoning gives the impression that the user is on the right track. Then, the length of the intention list is checked to elaborate more on whether the user follows one specific dish or there are still multiple possibilities. For the cases in which the goal is in the intention list, the robot gives feedback type of suggestions that give the information about the status of the action. The action represents the selected ingredient and is denoted by < *a* > . The action status could be *True* or *False* depending on whether the played action complies with the goal’s recipe. For example, say *Fois Gras Toast* is assigned as a target dish, and the user has already collected *foie-gras* and *truffle*. The recognized intention is *Fois Gras Toast*. Now the user collects *butter*: this action is *False* because collecting *butter* does not comply with *Fois Gras Toast*’s recipe since the recipe does not include butter. Therefore, the instantiated interaction will look like “*You lost a bit. You should remove butter.*” Here, it is interesting to note that such feedback was not requested by the user. Therefore, from the user’s perspective, it is a proactive action, as the robot is acting by itself by anticipating the future situation.

In phase 2 (see Figure 2B), it is crucial to keep up with the user to assist the user in accomplishing the user’s goal. The difference from phase 1 is that the robot is unaware of the goal: the user chooses it. The robot uses intent recognition to predict the goal of the user. The rules of the proactive suggestion focus more on the user’s consistency than on assisting. That is why the current intention list (which is the intent as shown in Figure 2B) and the previous intention list (which is the *pre*_*i*_ *nt* as shown in Figure 2B) are used for reasoning. After updating the intention list, it is checked whether or not the user’s intention is a specific dish. This means the length of the intention list is equal to one; therefore, a single intent is recognized. This case is treated similarly to a supposed target goal. That is why the status of the action is checked, as explained for the similar situation of Phase 1. If there is no specific intent, the system tries to lead the user by suggestions. The reasoning about suggestions starts with checking if there is any intent in the intention list or not. If the length of the intention list is equal to zero, the system tries to lead the user by suggesting the most frequent ingredient. If there is an intention which means the length of the intention list is non-zero, the length of previous intent is checked to be equal to one to determine if the user had a goal. In that case, the suggestion instantiates for explaining its reasoning and objectives of the previous goal. For example, in this situation, the robot said, “*I thought you were selecting ingredients for Fois Gras Toast so you should select truffle*.” Otherwise, the suggestion relates to the most popular element in the list.

### 3.3 Implementation Details

**FIGURE 3:**
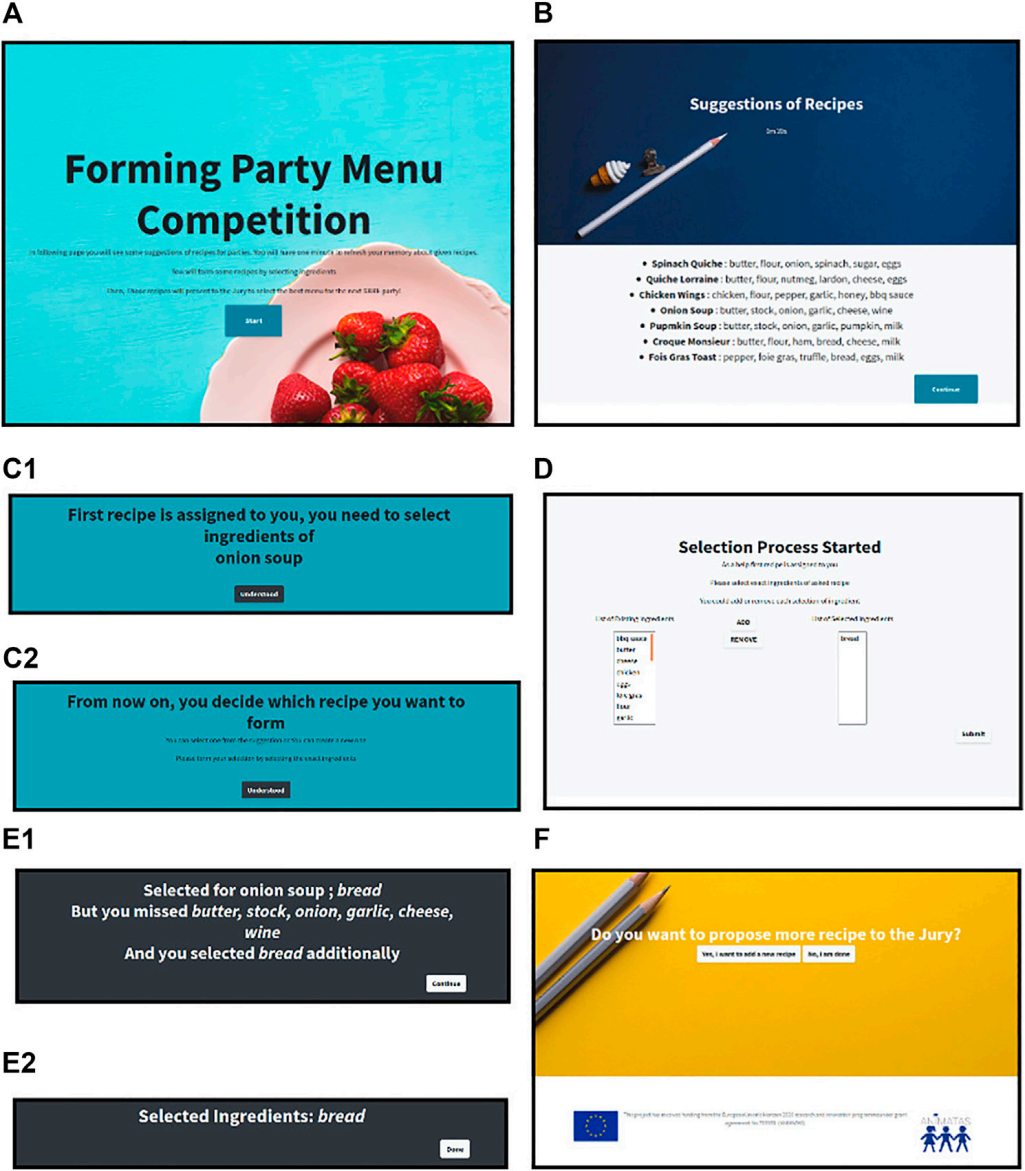
GUI of cooking recipe task; diagram combines the pages of the web-based task application. After the robot connected—additional pages omitted, **(A)** is welcoming the user and explaining about the aim of the task. **(B)** is only visible for 1 min, and it presents the examples of recipes. **(C)** is changed according to phases of the task, (C1) is phase 1, where a dish is assigned, and (C2) is phase 2, where the participant has freedom of choice. **(D)** is the page where phases of the task occur, where the robot proactive behavior is activated. It is identical for all phases. **(E)** is the result pages after each selection process. (E1) has a specialized view for phase 1 which provides more information. (E2) is the result page for phase 2 which only shows the recent information of selection. **(F)** is the page that comes after phase 2 and lets the participants continue to create more dishes or finalize the task.

The Pepper humanoid robot [description can be found in the work of Pandey and Gelin (2018)] interacted with the participants during the experiment. The robot followed the actions of the participants from a web-based interface and instantiated interactions from the Android application of the robot. The task was presented on the laptop with a web-based interface. The participant can only take actions and decisions with the laptop. The graphical user interface (GUI) that the participants faced is shown in Figure 3. The connection between the robot application and the web application is made using a Firebase database (Moroney and Moroney, 2017).

The interaction flow is shown in Figure 1. The diagram shows the combination of web-based task flow, the robot interruptions, and the participant’s actions. The task and behavior system are separated from each other to divert the participants’ focus from the robot to the task.

## 4 Evaluation

The in-person experiment was designed and conducted at the SoftBank Robotics Europe facility.

### 4.1 Participants

A total of 30 participants (11 female and 19 male, average age 32.23, standard deviation 6.76) participated in this experiment. All of them were employees of SoftBank Robotics Europe, Paris. They had some experience with the Pepper robot. However, they had different backgrounds: technical (hardware and software) and non-technical (marketing, communication, and welcome desk). The participants were also fluent in the language of the experiment: English. All participants gave their consent and signed a form giving permission to use and share their anonymous data for scientific purposes.

### 4.2 Hypotheses

We aim to study how the robot’s creativity (which is instantiated through the proactive interaction) affects 1) the perceived creativity of the robot and 2) the creative process of the human during an HRI task scenario. Recall that creativity is seen as bringing novelty, and proactivity is anticipatory behavior aiming to help in the task. Therefore, we developed the following hypotheses to study their relation and effects on the user’s perception.

H1: Proactivity and perceived creativity of the robot; the proactivity of the robot behavior will affect the perceived creativity. Proactivity in the robot behavior and its perceived creativity are related.
H2: Proactivity and the user’s creativity; there exists a link between the robot’s proactivity and the resulting creativity in the user (measured by the novelty of the products that the user creates in the HRI task). That is, proactivity of the robot and the facilitated creativity in the user are potentially related.
H3: Proactivity and goal achievement; there is a relationship between the robot’s proactivity and the success of the HRI task. That is, the proactive behavior of the robot can help to achieve the goal of the task.
H4: Proactivity level and user perception; different levels of proactivity of the robot will have different user experiences on the perceived attributes, including perceived and facilitated creativity.

### 4.3 Study Design

A between-subjects study was conducted with one independent variable, the proactive behavior of the robot, which has three conditions: *high*, *medium*, and *no proactive*. The different conditions of proactive behavior aim to change the frequency of exhibiting proactive interactions. Under full proactive conditions, it is expected that the robot will provide feedback after each action of the user. On the other hand, under no proactive condition, the robot is not providing any feedback. An intermediate condition (medium proactivity) is detailed below, along with details of the other conditions. The robot also talks at the start and between each phase of the task. Participants were randomly assigned to different conditions.

### 4.4 Conditions

The robot followed the general flow of the interaction with the participant, as shown in Figure 1. The main aim of the added interactive behavior is to balance the frequency of the robot’s talk between different conditions. In a between-subject study, the participants only interacted with one of the conditions. Three different conditions of the robot’s interactive behaviors are instantiated for this experiment. These conditions are as follows:

**Condition 1: no proactive behavior.** The robot does not provide any explicit or implicit directions to the user in terms of the status of the action. After each step of the ingredient selection process, the robot simply utters, “oh^1^.” We choose “oh” because it is a “knowledge state marker,” that is, when “oh” is uttered by the robot, it informs the user that the action they have undertaken is understood by the robot but does not give (either positive or negative) feedback on this action. Thus, as a knowledge state marker [as described in Heritage (1984)], “oh” is used as a neutral token to acknowledge to the user that their action has occurred.
**Condition 2: medium proactive behavior.** Under this condition of the experiment, the robot provides communicative proactive action at every third action of the participants and utters “oh” in other steps. The frequency of interventions is decided based on the approximate number of actions played in each phase. If everything goes well, the participants need to play six actions to accomplish the goal. It is decided that the robot acts at least every third action to (at minimum) have support at half of each phase. The proactive actions are instantiated through a response trigger mechanism described in Section 3.2.
**Condition 3: high-level (full) proactive behavior.** Under this condition, the robot instantiates and provides communicative proactive action after each action of the participants.

Thus, the kind of information the robot provides under medium and high proactive conditions is related to the ingredients selected by the user for a dish. At the end of each phase of the task, which is supposed to result in a recipe, the robot provides a summary of the selections. The response of the robot is instantiated by a matching mechanism using the database of known recipes, their ingredients, and the selection of ingredients by the user. If the participant created a new recipe (mainly by selecting a novel set of ingredients) that the robot could not find a matching recipe for, the robot asked the name of the potentially “new” recipe of selected ingredients to use this information for interaction purpose.

### 4.5 Setup

**FIGURE 4:**
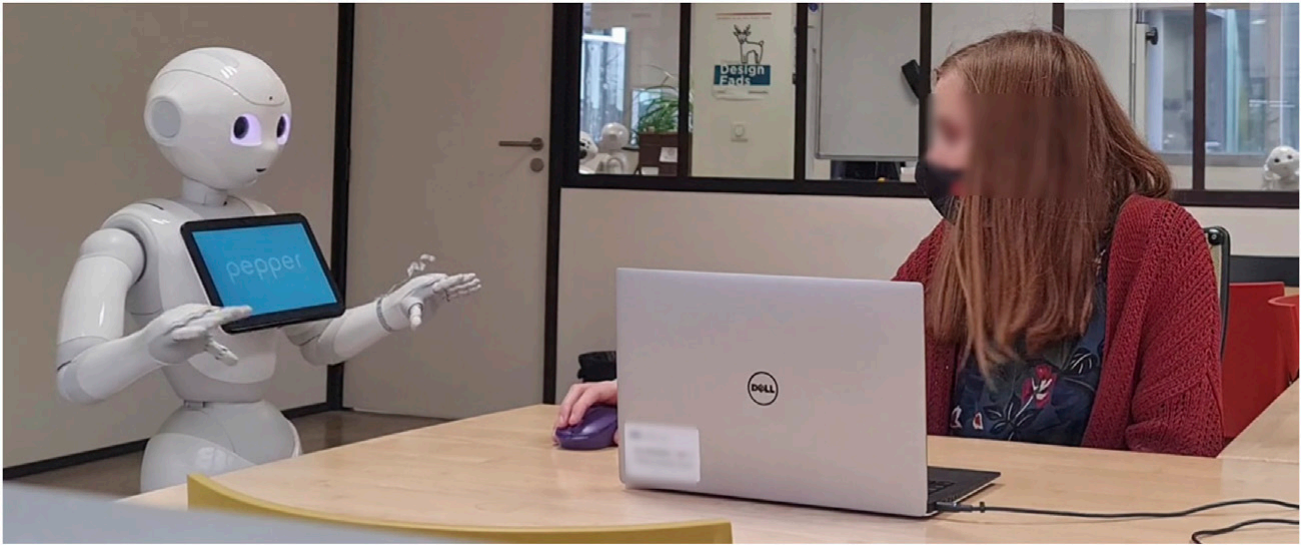
Set up of the experiment. Participant sits in front of a laptop and the robot is placed next to the user. They share the space as the robot is looking at the screen over the participants.

The experiment was conducted in the various meeting rooms of SoftBank Robotics Europe. The experimental setup is shown in Figure 4. The participant sat in front of a laptop to get engaged in the task. A Pepper robot was placed relatively to the left or right of the user. Participants manipulate the task environment on the screen of the laptop through a mouse or track pad. A self-report questionnaire is attached to the task and automatically pops up once the task is over. As a part of COVID 19 guidelines, all equipment was sanitized before and after each session. Participants were left alone in the room with the robot during the study.

### 4.6 Procedure

Procedures for all conditions are identical except the robot’s interruption frequency during the execution of the phases of the task. After signing the informed consent form, participants are informed about the experiment. Participants were given the choice of suggesting as many recipes as they wished for the upcoming hypothetical company event. The way to suggest is by using the online platform. They were informed that the online tool would guide them on how to proceed. Sample recipes were given to remind them how ingredients may be used. They could list as many recipes as they wanted while the Pepper robot accompanies them. They were reminded to be aware of the existence of the Pepper robot. Then, the experimenter left the room. Each participant interacted with one condition of the proactive robot behavior (condition 1: no proactive, condition 2: medium proactive, and condition 3: high proactive), which was assigned randomly and maintained during both the phases of the task. As a result, each participant can generally work on two kinds of dishes: one that is assigned to them and one that they created. Participants were also allowed to proceed without selecting any ingredient by submitting the result without collecting any ingredients at the execution of the phases in page (in Figure 3D). The proactive robot behavior is initiated depending on the robot’s knowledge. In this experiment, the task space and the participant’s action in the task space were used to enrich the knowledge of the robot. The robot stayed ignorant of the other possible actions from the participant or the shared environment. After each participant had completed the task, the self-report questionnaire was submitted. The self-report questionnaire is attached to the task interface. It automatically pops up when the task has been completed. After the participants completed the task and the self-report questionnaire, the experimenter came back to the room for a small interview.

### 4.7 Measurement

Different evaluation metrics are used to investigate different aspects of proactivity and creativity. Our measures are divided into three sections to assess the following:

Creativity of the user; to define and evaluate the participants’ creativity, metrics were inspired by divergent thinking. Thus, the creative thinking of the user often links with divergent thinking tests. Traditional methods of scoring divergent thinking (i.e., fluency, originality, and flexibility) are the most used methods for assessing the potential of creativity (Runco, 1992). In this study, we created an assessment influenced by the Torrance Test of Creative Thinking (Torrance, 1974) by focusing on fluency in the task—*How many dishes were achieved?—*and originality—*How many new dishes were created?*. These two scores are used to measure the creative thinking of the user. The total number of dishes is summed at the end of each task. It included phases 1 and 2 and repetitions of phase 2. The number of new dishes is the count of all dishes created in phase 2 and repetitions of phase 2. Dishes in the list of ingredients that have the same recipe as dishes in the recipe list are extracted.

Creativity of the robot; to assess some creative aspects of the robot’s behavior, a different self-report questionnaire was used to assess the participants’ perception of the robot. The questionnaire is a combination of different sections to assess demographic information, participants’ personality and creativity using a Likert Scale, acceptance of social robots from the ALMERE questionnaire (Heerink et al., 2010), comprehensive impression of user experience from the User Experience Questionnaire (UEQ) (Schrepp and Thomaschewski, 2019), and some specific questions directly related to engagement, proactivity, task, and overall interaction. In this study, we did not include all the scales from the questionnaire, such as ALMERE and UEQ. Instead, we included the scales that could be applicable to the defined situation such as perceived adaptivity, perceived enjoyment, attitude, perceived usefulness, trust, and dependability. The scales assess the participants’ perception about the robot’s creativity on generating proactive actions that are task-oriented.

Effect of proactivity; to assess the effects of different conditions of proactive behavior on the task, we check the success rate of phase 1. In phase 1, a random dish is assigned to the participants, and it is expected of the participants to select the exact ingredients which were shown to them earlier. We also analyzed the time that they spent during the selection process in phase 1. The spent time is calculated by the time that passed since the user started to select ingredients until they submitted their selection by clicking the submit button.

## 5 Results and Discussions

This section presented an evaluation for our four hypotheses (outlined in Section 4.2). We take the user study from 30 participants, 10 for each condition (no, medium, and high proactive behavior of the robot). We conducted a one-way analysis of variance (ANOVA) in each of our hypotheses to see if they are different for the three conditions: no (*n* = 10), medium (*n* = 10), and high (*n* = 10) proactive behavior with a *post hoc t*-test to compare differences in paired conditions. To test our hypotheses, recall that we use a combination of qualitative and quantitative measures (as is further illustrated in the following sections). For the qualitative data, we analyze the results of the questionnaire as the post test given to the participants. For quantitative data, we use the meta data generated from the results of the task (such as the number of times a dish was created). The data are presented as the mean and the standard deviation.

Before conducting the ANOVA test, we check that the following assumptions are not violated: 1) no significant outliers, 2) test for normality (by Shapiro–Wilk’s test), and 3) homogeneity of variances^2^ (by Levene’s test). We do not check for the independence of observations, as each participant belongs only to one condition/group. For the test to detect outliers, we check outliers for our quantitative data collected, but we keep the data since we manage to figure out the reason for the outlier. That is discussed in the following section. However, the qualitative data outliers are subjective reports and are essential for our analyses (e.g., how was the perceived adaptivity of the robot?). However, we do report this range of differences in the user’s opinion. The results are shown according to each hypothesis in the following sections. The significance of *p* is denoted by stars *, from high impact (***p < 0.001) to low impact (*p < 0.05), and nonsignificance is denoted by (ns *p* > 0.05). Qualitative observations are discussed in the following section, along with various attributes and pointers for further investigation.

### 5.1 Proactivity and Creativity

The *Novelty* scales of UEQ (User Experience Questionnaire) measures how much the design of the robot’s behavior is perceived as creative. The user is asked to rate from dull to creative, with the statement “*In my opinion, the idea behind the robot’s behavior and design is –*” on a 7-point scale (-3: dull to 3: creative). We conduct a one-way ANOVA test to see if the perceived creativity of the robot was different in our 3 group conditions. We run tests before conducting the one-way ANOVA test on the dependent variable (creativity) to check that the assumptions are met. We only have one outlier which is not extreme; one user in the medium condition rated the perceived creativity of the robot as dull ( − 1) compared to the *MEAN* = 1.2 of the group. The variable was normally distributed (*p* > 0.05) for each group, as assessed by Shapiro–Wilk’s test of normality. We can assume the homogeneity of variances in the different proactive conditions (*p* > 0.05 by Levene’s test).

**FIGURE 5:**
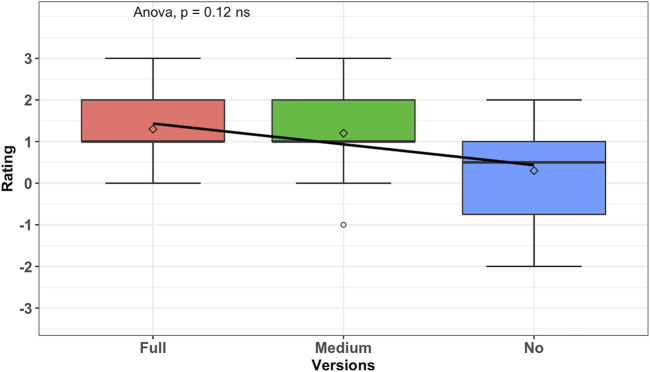
Analysis result of novelty from UEQ; the graph is scaled on positive as creative and negative as dull. It shows that in each version of proactive behavior, the robot rated as creative; however, there is a visible difference on the mean from full to no proactive behavior.

The results of the one-way ANOVA test on perceived creativity of the robot and our 3 conditions are given as (*ns*), *F*(2, 27) = 2.28, *p* = 0.12, *ges* = 0.14, as presented in Figure 5 (where *F* is the result of the test, and *ges* is the generalized effect size). Given the value of *p*, we cannot conclude on the difference between the group conditions and the perceived creativity of the robot. As shown in the figure, the mean and standard deviation (SD) of the conditions are the following: high proactive condition with *MEAN* = 1.3, SD  = 0.90, medium proactive condition with *MEAN* = 1.2, SD  = 1.08, and no proactive condition with *MEAN* = 0.3, SD  = 1.27. What is interesting to see is that the means of all three conditions of the robot’s proactive behavior are perceived as creative (with values above 0 in the UEQ). The two levels of proactivity (medium–high) are perceived as more creative (*MEAN* = 1.2 and *MEAN* = 1.3, respectively) than the no proactive condition (*MEAN* = 0.3). In the case of the no proactive condition, the mean is close to zero with *MEAN* = 0.3*,* SD  = 1.27, suggesting that participants may not have been able to assign a clear verdict about creativity in the robot’s behavior. This shows that there is some perceivable difference to the user between the no proactive conditions and the proactive conditions, though not statistically significant according to the method used. Looking deeper into the generalized effect size, we see that *ges* = 0.14 (14%). This means that 14% of the change in the perceived creativity of the robot could be affected by the proactive conditions.

Thus, even without a statistically significant difference (according to this method), as part of an exploratory analysis, we looked at the means of each condition and effect size and found that there still could be links with the proactivity of the robot and the perceived creativity of the robot. It is plausible that the users hesitated to rate the robot’s creativity as dull so as to not seem harsh, given the positively skewed labels. During the post-experiment interviews, the participants indicated that the simple acknowledgment from the robot (the knowledge state marker “oh”) was seen as better than no acknowledgment at all. For future work, it may be better to consider no verbal feedback whatsoever from the robot to test no proactivity.

Additionally, we did not find a statistically significant difference between the two levels of proactivity according to this method (medium with MEAN = 1.2, SD = 1.08 and full with MEAN = 1.3, SD = 0.90). Therefore, it is too early to establish any relationship between the frequency of proactive behavior and the scale of perceived creativity in the behavior. Therefore, H1 is not completely supported, in the sense that both parts are not validated (H1: proactivity and perceived creativity of the robot; the proactivity of the robot behavior will affect the perceived creativity. Proactivity in the robot behavior and its perceived creativity are related). The exploratory results suggest that the proactive condition is affecting the perceived creativity of the robot. But we could find statically significant links between the perceived creativity of the robot and the proactive condition robots to establish any relation. That is why there is a need for further investigation in this direction.

### 5.2 Observed Creativity in the User

To further explore the factors associated with the user’s creativity, we conducted various analyses on the quantitative data from the study, such as the following: how many recipes were completed successfully? How many new recipes were created?

The design of the experiment was to encourage participants to complete at least one dish in phase 1 and then to complete or create at least one dish in phase 2. After that, participants were free to continue further iterations of phase 2 and complete or create more dishes. Participants can also skip a phase without completing or creating a dish.

**FIGURE 6:**
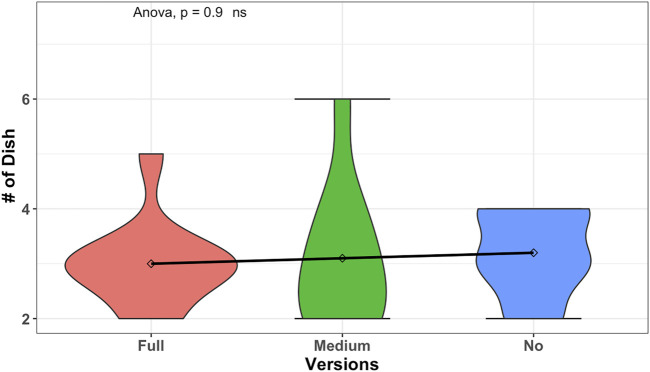
Comparison results of total number of completed or created recipes to the effects of different versions of robot behavior to be creative on completing or creating recipes by increasing number of recipes that is created in total is not shown as significantly changed.

We conduct a one-way ANOVA test to see if the total number of dishes that the user completed was different in our 3 group conditions. We run tests before conducting the one-way ANOVA test on the dependent variable (total number of completed dishes) to check that the assumptions are met. We have three outliers which are extreme; three users under the full condition completed (2,5,2) dishes compared to the *MEAN* = 3.0 of the group. The variable was normally distributed (*p* > 0.05) for medium and no proactive group conditions but was not normally distributed (*p* < 0.05) for the full proactive group condition, as assessed by Shapiro–Wilk’s test of normality. We can assume the homogeneity of variances in the different proactive conditions (*p* > 0.05 by Levene’s test). The results of the one-way ANOVA test to see if the total number of dishes that the user completed was different in our 3 group conditions are given as (*ns*), *F*(2, 27) = 0.10, *p* = 0.9, *ges* = 0.007 as presented in Figure 6. Given the value of *p*, we cannot conclude on the difference between the group conditions and the total number of dishes that the user completed. As shown in the figure, an almost straight trend line is observed between the conditions of the mean and standard deviation (SD) as follows: high proactive condition with *MEAN* = 3.0,  SD  = 0.81, medium proactive condition with *MEAN* = 3.1,  SD  = 1.28, and no proactive condition with *MEAN* = 3.2,  SD  = 0.78.

**FIGURE 7:**
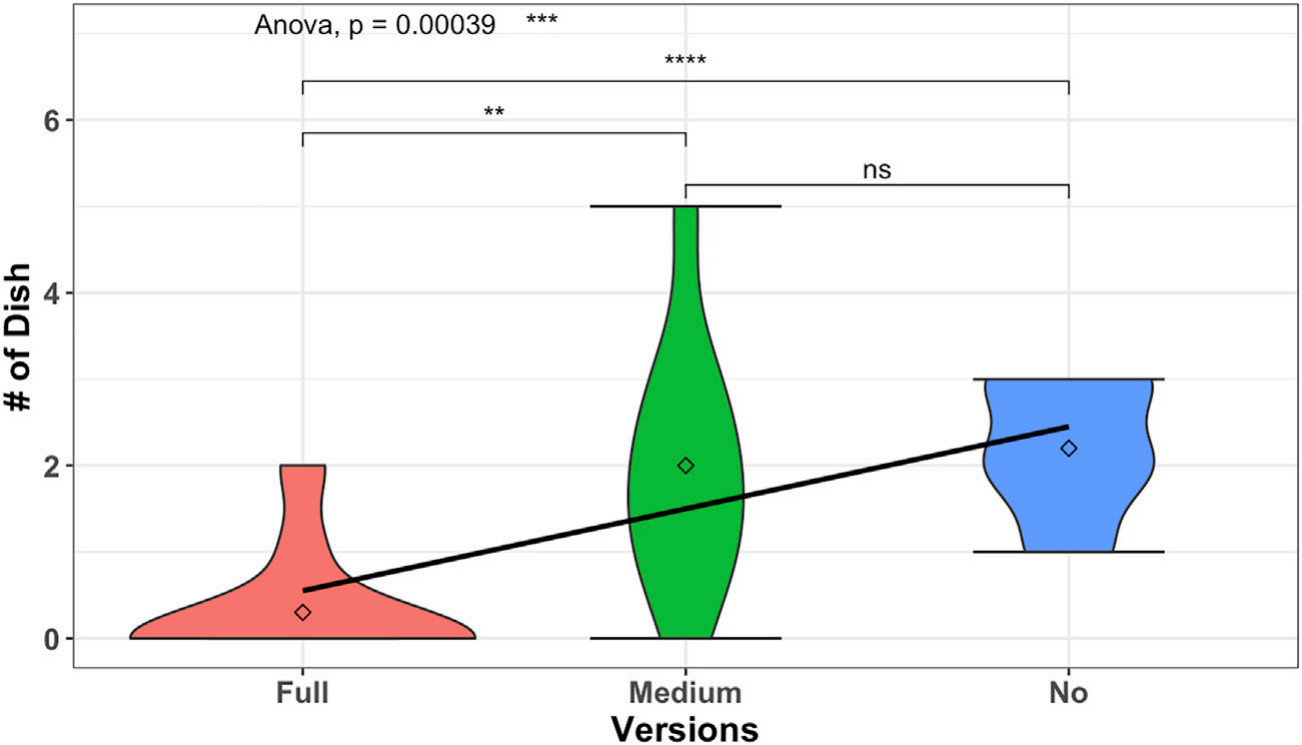
Comparison results of creating new recipe to the effects of different versions of robot behavior to be creative on creating new recipes. It clearly shows that the balanced proactivity (version of medium proactive robot) is supporting a greater number of new recipes to be created by the users.

However, we found interesting observations when we conducted a one-way ANOVA test to see if the number of new dishes created was different in our 3 group conditions. We run tests before conducting the one-way ANOVA test on the dependent variable (number of created new dishes) to check that the assumptions are met. We have two outliers which are extreme; two users under the full condition created (1,2) new dishes compared to the *MEAN* = 0.3 of the group. The variable was normally distributed (*p* > 0.05) for medium and no proactive group conditions but was not normally distributed (*p* < 0.05) for the full proactive group condition, as assessed by Shapiro–Wilk’s test of normality. We can assume the homogeneity of variances in the different proactive conditions (*p* > 0.05 by Levene’s test). The results of the one-way ANOVA test to see if the number of new dishes created was different in our 3 group conditions are given as (***), *F*(2, 27) = 10.62, *p* < 0.0003, *ges* = 0.44 as shown in Figure 7. This graph is related to phase 2 of the experiment, where the participants were free to converge toward a dish from the list or proceed toward creating a new dish. Given the value of *p*, creating new dishes had a statistically significant difference between the proactive behavior conditions. We followed up with *post hoc* tests (*t*-test) to multiple pairwise comparisons between groups. It can be seen from Figure 7 that there is a statistically significant difference between the no proactive condition and the full proactive condition with *p* = 0.000019(***) and between the no proactive and the medium proactive condition with *p* = 0.0045(**). There is no statistically significant difference (according to this method) between no and medium proactive conditions with *p* = 0.7(*ns*). Thus, it is observed that the number of new dishes which is created per person is significantly lower in full proactive conditions than in no and medium proactive conditions. Even in the no proactive condition with *MEAN* = 2.2,  SD  = 0.78 and the medium proactive condition with *MEAN* = 2,  SD  = 1.41, it shows that the number of new dishes which is created per person is lower in the medium proactive condition than in the no proactive condition. Hence, the analysis results support our hypothesis H2 (H2: proactivity and the user’s creativity). Once the robot is very proactive and heavily interrupting the user toward achieving a goal, participants can complete the task (as shown in Figure 6) but are not flexible and free enough to create new recipes, as shown in Figure 7, hence being less creative.

Another interesting observation is that the medium proactivity condition has the maximum number of created new dishes per person (at most 5), whereas for no proactivity, most of the participants stopped after creating a maximum of 3 new dishes. In medium proactivity conditions, the highest number of new dishes created per person is observed. This is fascinating and suggests a need for a balance. It hints that balanced proactivity could encourage prolonged creativity. It needs further studies to define the boundaries of the balanced proactivity.

In summary, there is no strong observation about different frequencies of proactive behavior on constructing a recipe. From Figure 6, it is shown from the bump in full and no proactive conditions that the majority of the people tend to create three dishes. So, the proactivity has not affected their motivation of creating recipes. However, it is also observed that when there is a space between interruptions, it kind of encourages the users to play more. On the other hand, when the robot proactively creates suggestions for the users, the users’ creativity decreases. The users tend to follow the robot suggestions and reduce their creativity process. As shown in the no proactivity case, since there is no help from the robot, users tend to be creative on constructing a recipe. However, in medium and, even heavily, in full proactive cases, when the robot is starting to help, the user’s flexibility for being creative seems to be reducing, as the users mostly go with the flow that the robot suggested. However, further study is needed to explain the reason for the changes in the user’s behavior.

### 5.3 Goal Achievement and Proactivity

**FIGURE 8:**
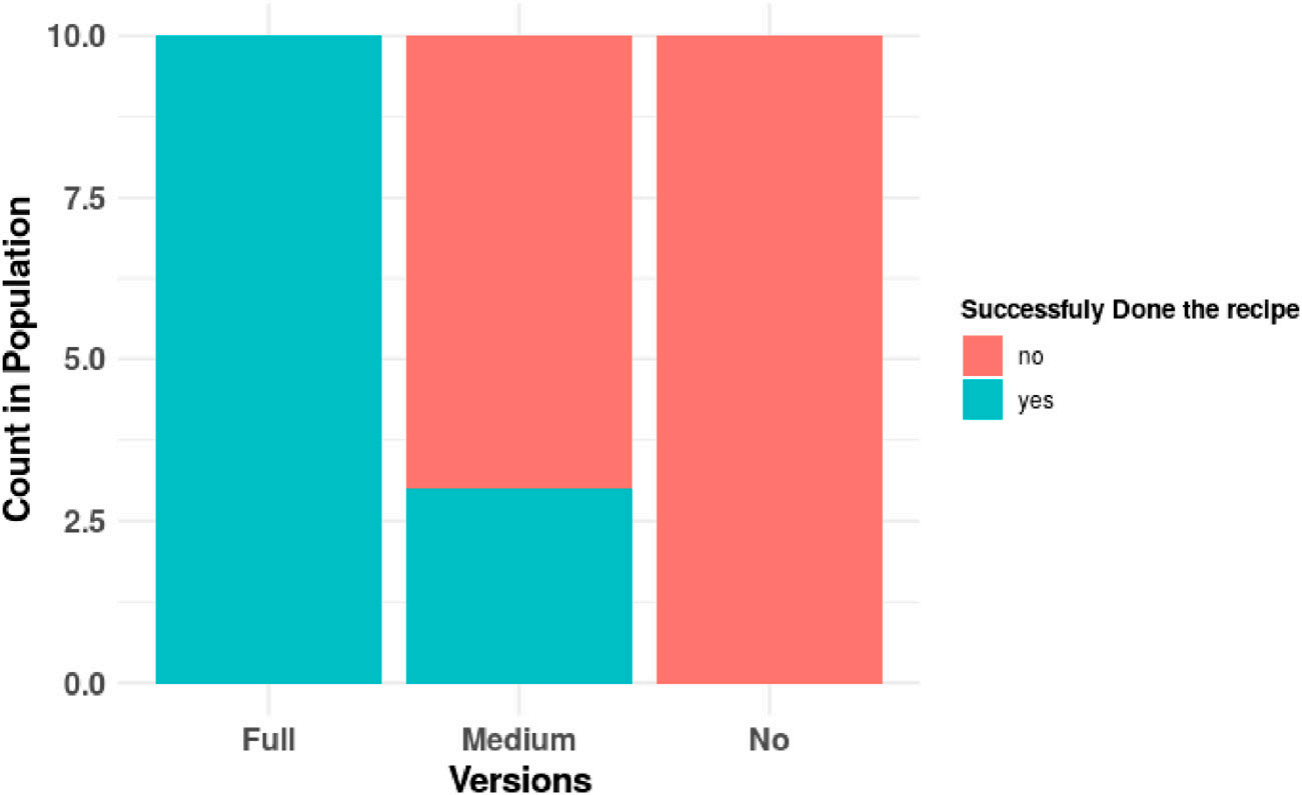
Distribution of successfully achieving to assigned dish; graph groups the counts of number of participants who achieved the assigned goal successfully during phase 1 to the experiment. It shows the absolute dominance of success in full proactive behavior and failure in no proactive behavior of the robot. In the medium proactive behavior of the robot, variations were observed to have success or failure.

**FIGURE 9:**
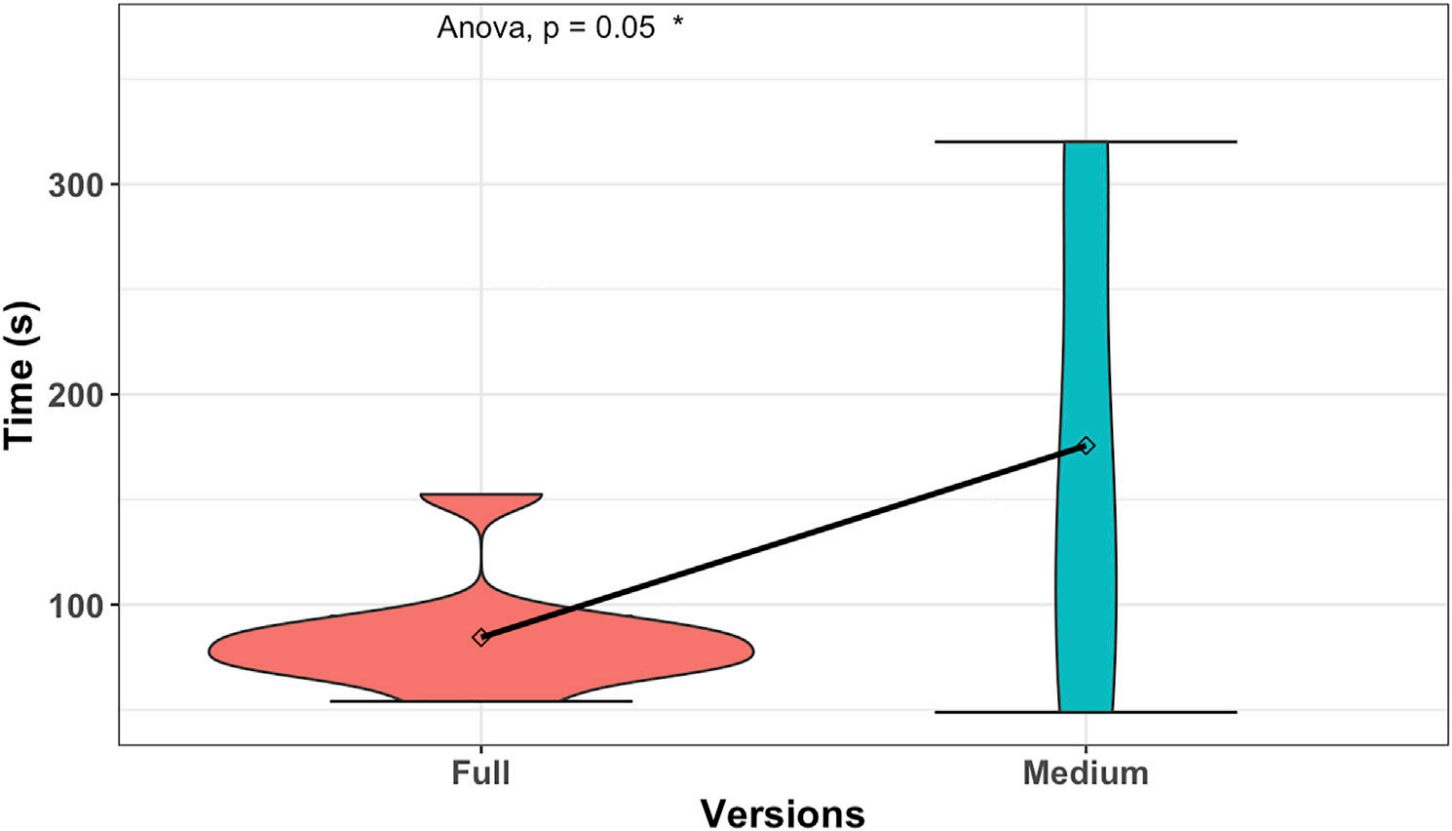
Detailed analysis of time spent on the correct result of different levels of proactive behavior; the graph aims to show the difference between how much time the users spend while reaching the correct results in phase 1.

To explore the benefits of proactive behavior on task accomplishment, we focus on phase 1 of the task and conducted the analysis of the comparison between each condition. How many times have recipes been done successfully? (see Figure 8) and how much time does the user spend while reaching the successful result? (see Figure 9) are used for analysis.

Figure 8 shows the successful completion of phase 1 of the experiment by the participants. As we can recall, in phase 1, the goal is assigned, and the target goal is known to the robot and the user. Hence, there is a joint goal to achieve. As we can see, all the participants exposed to the full proactive robot have successfully completed phase 1, whereas all the participants of the no proactive robot condition have failed. Furthermore, the failure rate was less in the medium proactivity condition, which shows 30*%* of success and 70*%* of failure. Pearson’s chi square test of independence is applied to statistically analyze the correlation between different proactive behavior conditions and successfully completing phase 1. The result shows that there is significant relation between different conditions and success, *X* ^2^(2, *N* = 30) = 21.44, *p* < 0.000022(***). This supports our hypothesis H3 (H3: proactivity and goal achievement; there is a relationship between the robot’s proactivity and the success of the HRI task. That is, the proactive behavior of the robot can help to achieve the goal of the task).

Furthermore, we conduct a one-way ANOVA test to see if the time spent between participants who achieved phase 1 successfully was different in group conditions. We run tests before conducting the one-way ANOVA test on the dependent variable (time spent) to check that the assumptions are met. We only have one extreme outlier; one user in the full proactive condition spent 152 s to reach success compared to the *MEAN* = 84.38 of the group. The variable was normally distributed (*p* > 0.05) for each (full and medium) condition. We can assume the homogeneity of the variances in different proactive conditions (*p* > 0.05 by Levene’s test). The results of the one-way ANOVA test on time spent between participants who achieved phase 1 successfully and our proactive conditions are given as (*), *F*(1, 11) = 4.84, *p* = 0.05, *ges* = 0.3 as shown in Figure 9. Given the value of *p*, we can observe a significant difference in time spent between participants who achieved phase 1 successfully. It should be noted that phase 1 does not include the creation of new recipes. Therefore, these two findings combined also indicate that participants are more successful and spend less time in reaching the goal, with robots having a higher frequency of proactive behaviors.

### 5.4 Proactivity Level and Effects on Perceived Attributes

**FIGURE 10:**
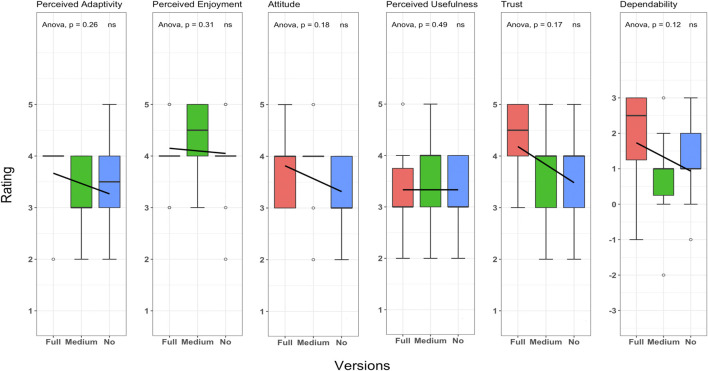
Analysis of questionnaire; the graph visualizes the united results of the questionnaire with ANOVA and *post hoc t*-test analysis. The scale is a 5-point Likert scale (ALMERE), except for Dependability, which was part of another subset of the questionnaire using a 7-point Likert scale (UEQ).

Figure 10 shows the overall impression of the participants about the robot’s behavior in different versions. Although we did not find statistically significant differences (according to this method) to reach any conclusion or establish any solid relation for each scale, we are pointing out some of the findings for further investigation. The summary of the analysis for each scale is as follows:

Perceived adaptivity is one of the scales of the ALMERE questionnaire that measures users’ perception of providing appropriate support by the robot. The user is asked to rate from 1: Do not Agree to 5: Totally Agree, with the statement “*I think the robot will help me when I consider it to be necessary*” on a 5-point Likert scale. We conduct a one-way ANOVA test to see if the perceived adaptivity of the robot was different in our 3 group conditions. We run tests before conducting the one-way ANOVA test on the dependent variable (perceived adaptivity) to check that the assumptions are met. We only have one extreme outlier; one user in the medium condition rated the perceived adaptivity of the robot as less not agreed (2) compared to the *MEAN* = 3.2 of the group. The variable was normally distributed (*p* > 0.05) for medium and no proactive conditions but was not normally distributed (*p* < 0.05) for the full proactive condition, as assessed by Shapiro–Wilk’s test of normality. We cannot assume the homogeneity of variances in the different proactive conditions (*p* < 0.05 by Levene’s test). The results of the one-way ANOVA test on perceived adaptivity of the robot and our 3 conditions are given as (*ns*), *F*(2, 27) = 1.43, *p* = 0.25, *ges* = 0.09. Given the value of *p*, we cannot conclude on the difference between the group conditions and the perceived adaptivity of the robot. As shown in the figure, the mean and standard deviation (SD) of the conditions are the following: high proactive condition with *MEAN* = 3.8, SD  = 0.63, medium proactive condition with *MEAN* = 3.2, SD  = 0.78, and no proactive condition with *MEAN* = 3.4, SD  = 0.96. In that sense, it is observed that participants found the full proactive condition of the robot the most adaptable. However, it is interesting to see the robot which did not give any suggestions be seen as more adaptable than the robot which is giving sparse suggestions (in the medium proactive condition). It might be because of various factors ranging from frustration of not getting enough suggestions (in case of the medium proactive condition) to the robot acknowledging behaviors being seen as completely supporting to the user action (in the no proactive condition). Therefore, this is another interesting direction for further investigations.

Perceived enjoyment is one of the scales of the ALMERE questionnaire that measures the level of enjoyment of the user while interacting with the robot. The user is asked to rate from 1: Do not Agree to 5: Totally Agree, with the statement “*I enjoyed the robot talking to me*” on a 5-point Likert scale. We conduct a one-way ANOVA test to see if the perceived enjoyment of the robot was different in our 3 group conditions. We run tests before conducting the one-way ANOVA test on the dependent variable (perceived enjoyment) to check that the assumptions are met. We have eight extreme outliers; four users in the full proactive condition, two of whom rated the perceived enjoyment of the robot as totally agree (5) and the other two rated as less not agree (2) compared to the *MEAN* = 4.00 of the group, and four users in the no proactive condition, two of whom rated the perceived enjoyment of the robot as totally agree (5), one of whom rated slightly agree (3), and the other rated less not agree (2) compared to the *MEAN* = of the group. The variable was normally distributed (*p* > 0.05) for full and no proactive conditions but was not normally distributed (*p* < 0.05) for the medium proactive condition, as assessed by Shapiro–Wilk’s test of normality. We can assume the homogeneity of variances in the different proactive conditions (*p* > 0.05 by Levene’s test). The results of the one-way ANOVA test on perceived enjoyment of the robot and our 3 conditions are given as (*ns*), *F*(2, 27) = 1.23, *p* = 0.30, *ges* = 0.08. Given the value of *p*, we cannot conclude on the difference between the group conditions and the perceived enjoyment of the robot. As shown in the figure, the mean and standard deviation (SD) of the conditions are the following: high proactive condition with *MEAN* = 4.00, SD  = 0.66, medium proactive condition with *MEAN* = 4.4, SD  = 0.69, and no proactive condition with *MEAN* = 3.9, SD  = 0.87. The results show that participants found the medium proactive behavior condition of the robot to be a more enjoyable companion. This might be because such behavior might not constrain the flow of the task very much with overload of suggestions or not seem engaged enough because of no suggestion.

Attitude is one of the scales of the ALMERE questionnaire that measures the user’s attitude toward the particular technology behind the version of robot behavior they have been exposed to. The user is asked to rate from 1: Do not Agree to 5: Totally Agree, with the statement “*The robot would make my life more interesting*” on a 5-point Likert scale. We conduct a one-way ANOVA test to see if the attitude toward the robot was different in our 3 group conditions. We run tests before conducting the one-way ANOVA test on the dependent variable (attitude) to check that the assumptions are met. We only have three extreme outliers; three users in the medium condition, one of whom rated the attitude toward the robot as less not agree (2), one of whom rated as slightly agree (3), and the other rated as totally agree (5) compared to the *MEAN* = 3.8 of the group. The variable was normally distributed (*p* > 0.05) for full and no proactive conditions but was not normally distributed (*p* < 0.05) for the medium proactive condition, as assessed by Shapiro–Wilk’s test of normality. We can assume the homogeneity of variances in the different proactive conditions (*p* > 0.05 by Levene’s test). The results of the one-way ANOVA test on attitude toward the robot and our 3 conditions are given as (*ns*), *F*(2, 27) = 1.82, *p* = 0.18, *ges* = 0.11. Given the value of *p*, we cannot conclude on the difference between the group conditions and the attitude toward the robot. As shown in the figure, the mean and standard deviation (SD) of the conditions are the following: high proactive condition with *MEAN* = 3.7, SD  = 0.67, medium proactive condition with *MEAN* = 3.8, SD  = 0.78, and no proactive condition with *MEAN* = 3.2, SD  = 0.78. The responses show that the medium proactive robot behavior is the most appreciated behavior.

Perceived usefulness is one of the scales of the ALMERE questionnaire that is another key aspect about the relevance of a particular behavior of the robot. The user is asked to rate from 1: Do not Agree to 5: Totally Agree, with the statement “*I think the robot can help me with many things*” on a 5-point Likert scale. We conduct a one-way ANOVA test to see if the perceived usefulness of the robot was different in our 3 group conditions. We run tests before conducting the one-way ANOVA test on the dependent variable (perceived usefulness) to check that the assumptions are met. We only have one outlier which is not extreme; one user in the full condition rated the perceived usefulness of the robot as totally agree (5) compared to the *MEAN* = 3.2 of the group. The variable was normally distributed (*p* > 0.05) for each condition, as assessed by Shapiro–Wilk’s test of normality. We can assume the homogeneity of variances in the different proactive conditions (*p* > 0.05 by Levene’s test). The results of the one-way ANOVA test on perceived usefulness of the robot and our 3 conditions are given as (*ns*), *F*(2, 27) = 0.73, *p* = 0.48, *ges* = 0.05. Given the value of *p*, we cannot conclude on the difference between the group conditions and the perceived usefulness of the robot. As shown in the figure, the mean and standard deviation (SD) of the conditions are the following: high proactive condition with *MEAN* = 3.2, SD  = 0.91, medium proactive condition with *MEAN* = 3.6, SD  = 0.84, and no proactive condition with *MEAN* = 3.2, SD  = 0.78. Again, the responses show that the medium proactive robot behavior is preferred by the users.

Trust is one of the scales of the ALMERE questionnaire that measures the user intentions to comply with the robot’s advice. The user is asked to rate from 1: Do not Agree to 5: Totally Agree, with the statement “*I would follow the advice the robot gives me*” on a 5-point Likert scale. We conduct a one-way ANOVA test to see if the trust toward the robot was different in our 3 group conditions. We run tests before conducting the one-way ANOVA test on the dependent variable (trust) to check that the assumptions are met. We do not have an outlier. The variable was normally distributed (*p* > 0.05) for medium and no proactive conditions but was not normally distributed (*p* < 0.05) for the full proactive condition, as assessed by Shapiro–Wilk’s test of normality. We can assume the homogeneity of variances in the different proactive conditions (*p* > 0.05 by Levene’s test). The results of the one-way ANOVA test on trust toward the robot and our 3 conditions are given as (*ns*), *F*(2, 27) = 1.92, *p* = 0.16, *ges* = 0.12. Given the value of *p*, we cannot conclude on the difference between the group conditions and the trust toward the robot. As shown in the figure, the mean and standard deviation (SD) of the conditions are the following: high proactive condition with *MEAN* = 4.3, SD  = 0.82, medium proactive condition with *MEAN* = 3.6, SD  = 1.07, and no proactive condition with *MEAN* = 3.6, SD  = 0.84. The results show that even the full proactive condition lead the user to rely more on the robot with the full proactive condition.

Dependability is one of the scales of UEQ (User Experience Questionnaire) that measures how much the reactions to the robot’s behavior are predictable. The user is asked to rate from dull to dependable, with the statement “*In my opinion, the reactions of the robot’s behavior to my input and command is –*” on a 7-point scale (-3:Unpredictable to 3:Predictable ). We conduct a one-way ANOVA test to see if the dependability of the robot was different in our 3 group conditions. We run tests before conducting the one-way ANOVA test on the dependent variable (dependability) to check that the assumptions are met. We have three outliers which are not extreme; two users in the medium proactive condition rated the dependability of the robot one as ( − 2) and one as dependable (3) compared to the *MEAN* = 0.80 of the group and one user in the no proactive condition rated the dependability of the robot as ( − 1) compared to the *MEAN* = 1.20 of the group. The variable was normally distributed (*p* > 0.05) for medium and no proactive conditions but was not normally distributed (*p* < 0.05) for the full proactive condition, as assessed by Shapiro–Wilk’s test of normality. We can assume the homogeneity of variances in the different proactive conditions (*p* > 0.05 by Levene’s test). The results of the one-way ANOVA test on dependability of the robot and our 3 conditions are given as (*ns*), *F*(2, 27) = 2.3, *p* = 0.11, *ges* = 0.14, (where *F* is the result of the test, and *ges* is the generalized effect size). Given the value of *p*, we cannot conclude on the difference between the group conditions and the trust toward the robot. As shown in the figure, the mean and standard deviation (SD) of the conditions are the following: high proactive condition with *MEAN* = 2.0, SD  = 1.33, medium proactive condition with *MEAN* = 0.8, SD  = 1.31, and no proactive condition with *MEAN* = 1.2, SD  = 1.13. The initial findings suggest that the participants listened more to the robot, which generated more advice than the full proactive condition of the robot.

It will be interesting to investigate further in these directions to find the factors behind these observations and to further explore the right level of proactivity for the interaction to be more enjoyable, adaptive, useful, and establishing the necessary trust and dependability at the same time.

Such differences in the perception of different attributes in different versions of robot behavior support our hypothesis H4: proactivity level and user perception different levels of proactivity of the robot will have different user experiences on the perceived attributes.

## 6 Discussion on Qualitative Observations

The interaction with the robot was not always so smooth. There were some problems related to the robot’s vocal feedback such as some participants confusing the verb “egg” with “ice.” So, they spent more time on understanding the robot’s suggestion.

There were some cases in which the dish’s name was the same as that known to the robot, but the participants selected different ingredients to create their own version of the dish. Those cases need to be investigated in future studies. However, it created an interesting interaction pattern as follows:

**Robot:** I thought you are selecting ingredients for < ‥*dish*‥ > but I don’t know this recipe.
**Robot:** Could you please tell me the name of it?
**Participant:** I know but this is my < ‥*dish*‥ > that’s why it’s different.

In the current analysis, if the dish’s ingredients are different, it is classified as a new dish since creativity assessment depends on knowledge. We classify the novelty of recipe creation according to what is provided by the task and what is known from the robot. It is important not to forget that the robot could only help with the limitation of its knowledge.

Participants of experiments are the employees of SoftBank Robotics Europe. They had experience with the Pepper robot. However, their background is mixed between technical (hardware and software) and non-technical (marketing, communication, and welcome desk). Nevertheless, this can introduce bias in terms of a more positive attitude toward the robot. In the future, we aim to experiment with more diverse users, hopefully, once the Covid-19 restrictions are over.

Some participants listened to the robot’s feedback for the first phase but not very much during the second phase onward. Later, they stated, “*I knew what I was doing, so I did not listen to the robot’s advice*” or “*I already asked the robot for help, it did not help me. Then, it offered some help. This time, I refused it.*” Such feedback indicates that in addition to considering the goal and future needs, the robot should also incorporate social signals and some aspects of reactiveness while generating its proactive behavior. It will be interesting to explore such factors and develop an inclusive computational model for behavior generation.

The effect of agency and embodied presence of the robot was observed strongly. For example, some participants perceived the “oh” response as positive, while others perceived negatively as respond proved as a neutral response to not reflect any opinion. It is expected from the extraction of previous research (Heritage, 1984) that involuntary interruption means anything. Some participants also think that the robot is enjoying the selections, so they continue to create a recipe process. Participants were so eager to get any reaction from the robot that they tried to put different naming. Some participants also played tricks to validate their perception about “oh” behavior at a medium proactive condition of the robot. This suggests that even some involuntary interruptions will keep participants motivated in a task, which might contribute to their prolonged creative “experiments.” We believe that these differences in perception are related to participants’ tendency to extract the meaning of each noise from the robot. It is not incontrovertible of the familiarity of participants with the robot. This is another exciting direction for further studying the connection between robots’ behavior and its effect on creativity in the user.

It is observed that sometimes the ingredients were limited to create an entirely new recipe. In those cases, the task-oriented proactive verbal communicative actions of the robot also confused some participants, as they stated that “I was not sure should I create a new recipe or try to create one of the given ones.” Also, as some of the participants mentioned that they were not very good with cooking and recipe knowledge, that might be contributing to participants following the feedback from the robot. Such observations need further investigation on understanding the more in-depth relation between proactivity and creativity in an open-ended and domain-independent scenario.

## 7 Conclusion

This study attempts to explore the behavioral aspect of creativity in robots in the context of human–robot interaction. We hypothesized the dimension of bringing novelty in behavior by proactive actions by letting the robot initiate a suggestive interaction for a task that humans are supposed to do. We have presented the creative cooking experiment and analysis with the humanoid robot, Pepper. As this is an exploratory study, the preliminary finding hints toward the proactive behaviors of the robot somewhat affecting the perceived creativity of the robot. We have also provided pointers such as proactive behaviors not only leading users but also helping to keep achieving the goal of the task. We have shown that different levels of proactive behaviors have different effects and relations with various aspects of perceived attributes. To our knowledge, this is the first study of its kind on understanding the creativity and proactivity aspects together in a human–robot interaction context, from the perspective of achieving a goal and from the perspective of supporting creativity in the user. We have discussed and pointed out various aspects needing further investigation to strengthen our knowledge in this domain, including the finding that there seem to be trade-offs to find the right level of proactivity that will help to achieve the goal but leave space for the user to be creative, which we think is very important for the real-world deployment of social robots in day-to-day tasks and companionship.

### 7.1 Limitation of the Study

Due to the COVID-19 lockdown in France, it was not possible to conduct a physical experiment with potential end users. That is why we conduct a physical experiment with SoftBank Robotics Europe employees who have special allowance to enter the working area.

## Data Availability

The raw data supporting the conclusions of this article will be made available by the authors, without undue reservation.

## Footnotes

1 In the implementation using a Pepper robot, the exact token used was “oo,” as “oh” sounded unnatural given Pepper’s text-to-speech component.

2 Based on the practices as outlined in this resource https://www.datanovia.com/en/lessons/anova-in-r/#check-assumptions.
